# The contradictory role of branched-chain amino acids in lifespan and insulin resistance

**DOI:** 10.3389/fnut.2023.1189982

**Published:** 2023-06-20

**Authors:** He Yao, Kai Li, Jie Wei, Yajun Lin, Yinghua Liu

**Affiliations:** ^1^The Key Laboratory of Geriatrics, Beijing Institute of Geriatrics, Beijing Hospital, National Center of Gerontology, National Health Commission, Institute of Geriatric Medicine, Chinese Academy of Medical Sciences, Beijing, China; ^2^Department of General Surgery, The First People’s Hospital of Taian, Taian, Shandong, China; ^3^Department of Nutrition, National Clinical Research Center for Geriatric Diseases, The First Medical Center of Chinese PLA General Hospital, Beijing, China

**Keywords:** branched-chain amino acids, aging, insulin resistance, lifespan, longevity

## Abstract

Branched-chain amino acids (BCAAs; a mixture of leucine, valine and isoleucine) have important regulatory effects on glucose and lipid metabolism, protein synthesis and longevity. Many studies have reported that circulating BCAA levels or dietary intake of BCAAs is associated with longevity, sarcopenia, obesity, and diabetes. Among them, the influence of BCAAs on aging and insulin resistance often present different benefits or harmful effects in the elderly and in animals. Considering the nonobvious correlation between circulating BCAA levels and BCAA uptake, as well as the influence of diseases, diet and aging on the body, some of the contradictory conclusions have been drawn. The regulatory mechanism of the remaining contradictory role may be related to endogenous branched-chain amino acid levels, branched-chain amino acid metabolism and mTOR-related autophagy. Furthermore, the recent discovery that insulin resistance may be independent of longevity has expanded the research thinking related to the regulatory mechanism among the three. However, the negative effects of BCAAs on longevity and insulin resistance were mostly observed in high-fat diet-fed subjects or obese individuals, while the effects in other diseases still need to be studied further. In conclusion, there is still no definite conclusion on the specific conditions under which BCAAs and insulin resistance extend life, shorten life, or do not change lifespan, and there is still no credible and comprehensive explanation for the different effects of BCAAs and insulin resistance on lifespan.

## 1. Introduction

**Figure 1:**
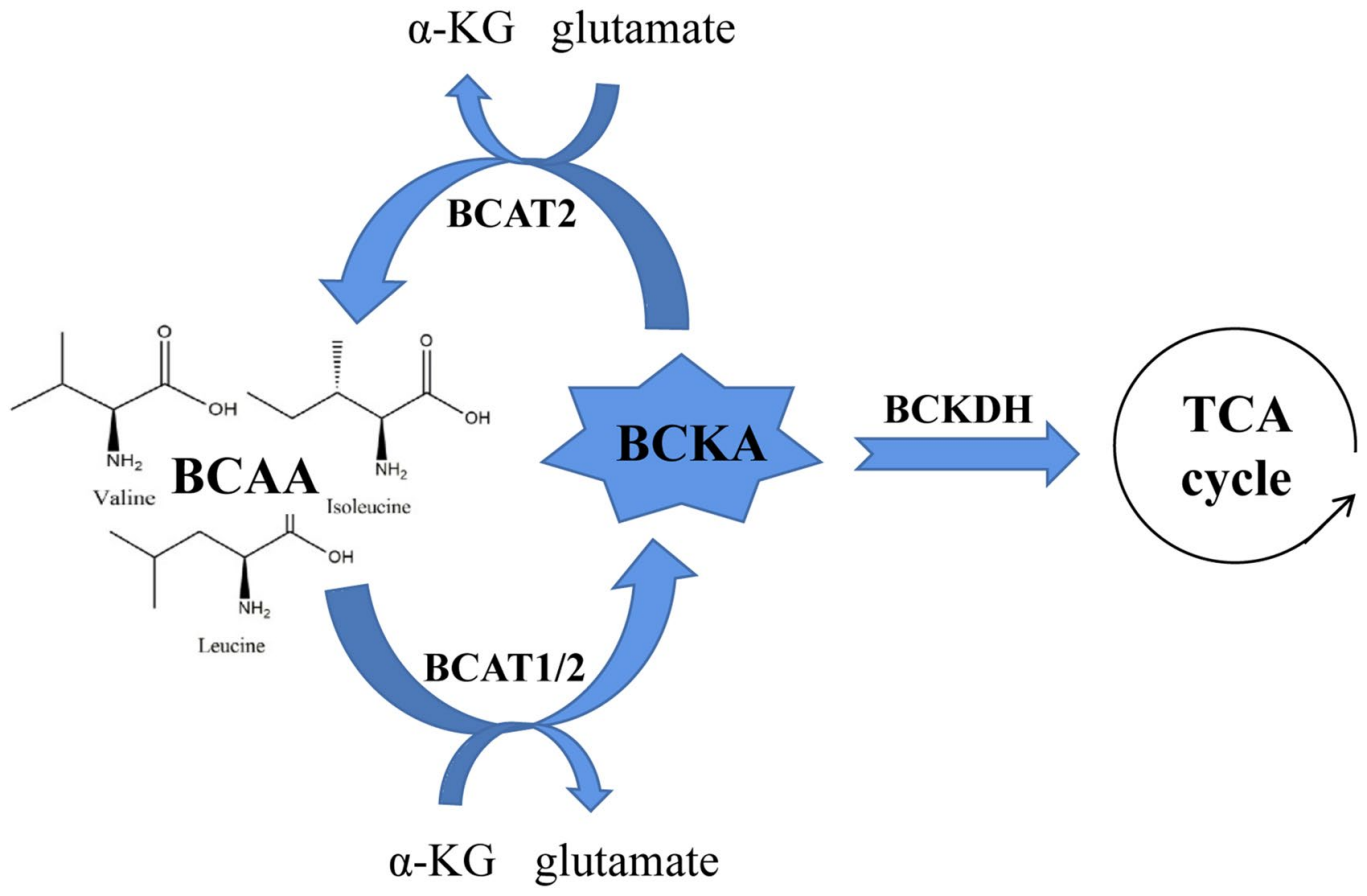
A brief schematic of BCAA catabolism and the enzymes that regulate this process.

Branched-chain amino acids (BCAAs), a general term for leucine, isoleucine and valine, are a common protein supplement (1). BCAAs play an important physiological role in regulating protein synthesis, metabolism, food intake and aging (2). Among them, BCAA circulatory levels are strictly regulated by catabolism, and BCAA abnormalities are associated with obesity, insulin resistance, type 2 diabetes, heart disease and cancer. All of the amino acids classified as BCAAs are catalyzed by branched-chain amino acid transaminase 1/2 (BCAT1/2) to form branched-chain ketoacid (BCKA), which is then oxidized by the branched-chain ketoacid dehydrogenase (BCKDH) complex to form the final metabolites. These metabolites enter the tricarboxylic acid cycle to generate energy. To maintain the catabolic cycle, BCAT2 reversibly catalyzes the initial steps of BCAA catabolism to regenerate BCAAs and α-KG (α-Ketoglutarate) (3, 4). Hence, we speculate that the increase in circulating BCAA levels caused by various diseases may be the result of BCAA metabolic disorder, which occurs by BCKA accumulation, changes in BCKDH activity, etc. (5) (Figure 1).

BCAAs are closely related to abnormal glucose and lipid metabolism, and studies on animal models have shown that dietary BCAAs are important inducers of insulin resistance (6), and limiting dietary BCAAs can help prevent insulin resistance (7). BCAAs can induce insulin resistance by promoting fat accumulation in muscles and transporting 3-hydroxyisobutyric acid (a catabolic intermediate of valine) across the vascular endothelium (8). Insulin resistance refers to a decrease in the efficiency of insulin in promoting glucose uptake and utilization and is associated with metabolic syndrome and type 2 diabetes mellitus (T2DM). Previous studies have linked aging to high blood pressure, high glucose levels and low insulin levels (9). Insulin resistance is an important cause and symptom of cardiovascular disease, T2DM and other diseases, and its occurrence causes great damage to organs (10). Insulin resistance in the aging process also causes sarcopenia (11). However, low levels of insulin resistance tend to contribute to longevity (12). It has been reported that low levels of insulin/IGF-1 signaling can prolong life. In *C. elegans* (*Caenorhabditis elegans*), mutations in the insulin/IGF-1 receptor gene DAF-2 or other genes in this pathway extend lifespan (13). In addition, in some insulin-related knockout mouse models, in which insulin resistance is induced in one or more tissues, a significant increase in the lifespan of mice can be observed (14, 15). All of these findings suggest that inhibition of insulin-related pathways promotes longevity. However, these contradictory reports raise a more important question: Is insulin resistance harmful or beneficial to humans?

To explore the above questions, scientists began to take life expectancy as an indicator and explored the relationship between BCAA intake and life expectancy. The results show that BCAAs are associated with longevity/aging and metabolic regulation through a variety of mechanisms. However, some studies suggest that BCAA restriction may be beneficial to longevity/metabolic health. High intake of red meat containing high levels of BCAAs may be associated with the promotion of age-related diseases (16). Other studies suggest that BCAA supplements may improve aging and prolong the life of middle-aged mice in multiple ways (17). This paradoxical finding is consistent with the contradictory relationship between insulin resistance and longevity. This information also suggests that BCAAs, insulin resistance and longevity may be closely correlated.

Therefore, this review summarizes and discusses the results of pairwise studies on BCAAs, insulin resistance and longevity and raises some questions. First, in terms of BCAAs and longevity, are BCAA-restricted and BCAA-rich diets good or bad for humans? Second, is insulin resistance induced by BCAAs beneficial or harmful? Third, under what circumstances does BCAA-induced insulin resistance occur? Fourth, does insulin resistance increase or decrease lifespan? This review provides an overview of recent studies and possible regulatory mechanisms from the above aspects. In addition, a new theory that insulin resistance is unrelated to longevity will be discussed in the following sections.

## 2. BCAAs and lifespan

The elderly population is increasing as a result of increased life expectancy, improved health status and improved medical services. The number of people aged 65 and over reached 617 million (8.5%) in 2015 and is expected to rise to 1.6 billion by 2050. Globally, from 1990 to 2017, life expectancy increased by 7.4 years, from 65.6 years in 1990 to 73.0 years in 2017 (18). While the average lifespan has increased, the corresponding healthy lifespan has not (19). Recent studies have shown that the average age of health is declining yearly, and nutrition appears to be an important factor in increasing healthy life expectancy. Disorder of protein metabolism due to aging is often accompanied by sarcopenia, which greatly reduces the quality of life of elderly individuals (20). With increasing age, human mitochondrial function gradually decreases through a decrease in mitochondrial DNA copy number and the ATP production rate (21). Dietary supplementation fortified with BCAAs may help to slow mitochondrial decay, improve the clinical situation of malnourished elderly patients (22), and increase healthy life expectancy, and this approach is more effective than simple dietary recommendations (19). Its mechanism may be related to increases in mitochondrial biogenesis and antioxidant capacity in the muscle of aged rats induced by BCAA-rich foods. Studies have shown that BCAA supplementation in middle-aged mice significantly increased the occurrence of mitochondrial biogenesis in the heart and skeletal muscle and significantly improved the oxidative stress state in the heart and skeletal muscle by increasing the activity of mTORC1. Following these alterations, the lifespan of middle-aged mice was extended by 12% (17).

On the other hand, supplementation with BCAAs can also improve aging by promoting muscle protein synthesis and iron content (23), and BCAA supplementation significantly inhibits the decrease in serum albumin in elderly patients compared with that in young patients (24). In addition, BCAA supplementation can promote wound healing, reduce the occurrence of kidney and liver diseases with age (25), and reduce inflammatory markers in patients with chronic heart failure (26).

Furthermore, after the addition of BCAAs, the structure of the intestinal flora in mice was changed, and the rate of changes in the intestinal flora in mice due to age was slowed. This finding suggests that BCAAs affect the intestinal flora and intestinal metabolism. The concentration of lipopolysaccharide binding protein in the serum of BCAA-group mice was low. These changes indicate reduced antigen load in the host intestinal tract. It has been suggested that adding BCAAs to the diet can improve the intestinal flora and promote healthy aging (27).

**Figure 2:**
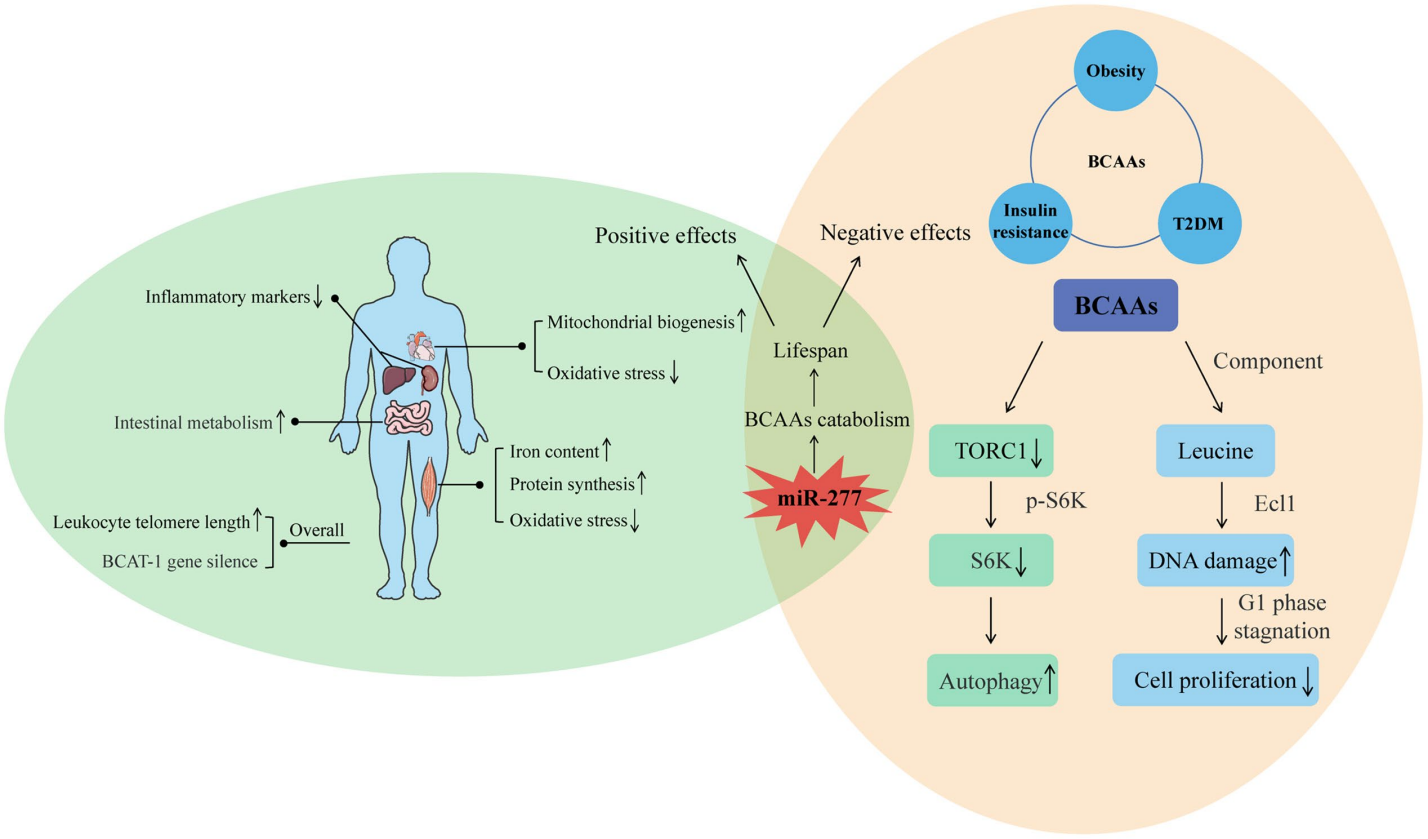
Positive and negative effects of BCAAs on the body. For the positive, the intake of BCAAs can improve the function of muscle and heart, reduce the inflammation of liver and kidney, and enhance intestinal metabolism. Moreover, increased endogenous BCAAs caused by BCAT-1 gene silence and increased leukocyte telomere length can also have a positive effect on longevity. For the negative, the effects of BCAAs on insulin resistance, obesity, and T2DM indicate their negative influence on lifespan. Furthermore, the molecular mechanism of BCAAs on shortened life is related to the autophagy induced by decreased TORC1, and the decreased cell proliferation induced by DNA damage. From the two sides, miR-277 seems a potential regulatory target whose expression level is associated with lifespan through adjusting BCAAs catabolism.

Endogenous BCAA accumulation also has a positive effect on longevity. In *C. elegans*, BCAT-1 gene silencing in wild-type adults increases endogenous BCAA accumulation and prolongs lifespan (28). In addition, in a population study, taking the long-lived village in East Asia as the latest research object shows that endogenous BCAAs have a potential protective effect on leukocyte telomere length and weakness and suggests a potential synergistic effect between BCAAs and leukocyte telomere length during healthy aging (29) (Figure 2).

High levels of BCAAs are often detected in the plasma of patients with T2DM and obese people (28, 30). However, considering the health benefits of BCAAs, the correlations between high levels of BCAAs and insulin resistance, obesity and T2DM seem contradictory. Studies have shown that an increase in circulating BCAAs was associated with an increasing risk of tissue damage both in mice, and maybe eventually lead to the mortality in mice, while BCAA restriction ranging from 50% (0.5 x BCAAs) to 85% can result in an additional increase in lifespan of Drosophila, and the amplitude of increase could reach about 10%. The possible mechanism by which BCAAs negatively regulate lifespan is by reducing TORC1 activity, reducing the phosphorylation level of S6K (S6 kinase), downregulating S6K mRNA translation, and inducing autophagy (31). BCAAs are effective activators of the TOR signal, and S6K is a direct downstream target of this signal. Research results show that overexpression of ARGK-1 can extend the life of *C. elegans* by activating the regulation of the energy sensor AAK-2/AMPK to RSK-1/S6K (32). Life expectancy was also increased by 9% in S6K knockout mice, suggesting that downregulation of S6K can prolong the lifespan of lower animals and mammals (33).

Studies on rapamycin-induced Drosophila autophagy (34) and dietary restriction-triggered *C. elegans* autophagy (35) have demonstrated that initiation of autophagy can lead to prolonged lifespan. In addition, adverse effects of leucine, the main component of branched-chain amino acids, on longevity have been reported. Leucine can induce senescence of MC3T3-E1 cells through DNA damage, which has a negative effect on the proliferation of bone cells. These results may provide new insights into previous studies on the supplementation of amino acids to promote bone health (36). Depletion of leucine prolongs the life of leucine-auxotrophic cells, leading to cell senescence and cell cycle stagnation in the G1 phase, which are dependent on genes in the Ecl1 family (37). These studies suggest that leucine, as one of the main components of BCAAs, may play an important role in the negative effects of aging.

In recent years, an increasing number of studies have begun to seek the best way to regulate lifespan through the study of branched-chain amino acid metabolism. Among them, BCAA catabolism regulated by miR-277 is the best way to study the regulation of BCAAs on lifespan. Overexpression and inhibition of miR-277 can shorten the lifespan, especially when consuming foods containing excessive protein. Expression of miR-277 reduced the metabolic capacity of BCAAs. The resulting increase in BCAA concentration may stimulate TOR kinase (38, 39), thereby shortening lifespan. Transgenic inhibition with the use of miRNA sponges also shortens the lifespan, especially on a protein-rich diet (40). Changes in miRNA expression in adult Drosophila showed that miR-277 was significantly downregulated in adult Drosophila, which was consistent with its effect on lifespan. Therefore, optimal metabolic adaptation and optimal longevity effects seem to be achieved by regulating BCAA catabolism by miR-277 (Figure 2).

In summary, the effects of BCAAs on longevity have many controversial consequences, and the possible mechanisms are complex. It is certain that the positive and negative regulation of BCAAs on longevity has a great relationship with the external environment, diseases and intake. For example, supplementing BCAAs under specific dietary restrictions can improve metabolic health, improve glucose tolerance, and reduce fat accumulation (41), while BCAAs in combination with a high-fat diet tend to lead to the development of obesity-related insulin resistance (42). The regulatory mechanism of BCAAs on longevity may be related to the regulation by miR-277 of BCAA catabolism.

## 3. BCAAs and insulin resistance

Many studies have confirmed that BCAAs may play a causal role in the pathogenesis of type 2 diabetes (43–45), and BCAAs contribute to the development of obesity-related insulin resistance in humans and rodents on a high-fat diet (HFD) (46). Studies of male rats and humans have shown that high-level BCAA intake is associated with high fat intake, leading to insulin resistance and glucose intolerance, which ultimately result in metabolic syndrome (42, 47). In obese mice induced by a high-fat diet, BCAA supplementation exacerbated obesity-related hepatic glucose and lipid metabolism disorders by weakening Akt2 signaling, thereby causing severe hepatic metabolic disorders and hepatic insulin resistance (48). Moreover, supplementation with BCAAs combined with a high-fat diet was associated with increased plasma BCAA concentrations in adult rats (42). Moreover, reducing BCAAs in the diet in this situation moderately improves glucose tolerance and reduces fat mass increase (49). Similarly, high BCAA levels in breast milk are associated with a high risk of insulin resistance in the children of obese women (50). A maternal high-fat diet alone or supplemented with BCAAs can impair the glycemic control of these children in adulthood. Male and female offspring fed high-fat diets containing BCAAs showed serious insulin intolerance and elevated fasting blood glucose levels, which might be related to the influence of the hypothalamic ER-α pathway (51). In addition, daily intake of BCAAs, namely, leucine, isoleucine, and valine, by mothers with gestational diabetes may also increase the risk of overweight and insulin resistance in their children (7). Other studies have not found an association between dietary BCAA intake and adult plasma BCAA concentrations (52, 53). In contrast, supplementing the maternal diet with BCAAs prevents age-related and diet-related insulin intolerance, thereby protecting offspring.

In healthy individuals, BCAAs appear to play beneficial roles, including reducing the risk of obesity, increasing muscle mass, potentially improving glucose sensitivity, and possibly exerting therapeutic effects in patients with cirrhosis and encephalopathy (54–57). However, high levels of circulating BCAAs in serum or plasma are thought to be associated with obesity and insulin resistance (58). Studies have shown that long-term exposure to high levels of BCAAs stimulates hyperlipidemia and obesity, which has a positive association with fasting glucose levels, LDL and triglyceride levels and a negative correlation with HDL-C and inhibits the TCA (tricarboxylic acid cycle) directly through BCKA accumulation (54, 59–61). When BCAA metabolic disorders occur, such as in metabolic syndrome, the catabolites of valine, isoleucine, and leucine may accumulate and produce negative metabolic effects. Furthermore, the risk of developing T2DM increases because of the genetic predisposition to BCAA metabolism impairment (62). Several studies have reported that plasma or serum BCAA levels can predict the development of T2DM (63).

BCAA metabolites are associated with the risk of obesity and insulin resistance. However, it is unknown which BCAAs, that is, leucine, valine or isoleucine, influence insulin resistance. Studies using diet deprivation—the complete removal of an amino acid from the diet—have found that eliminating leucine from the diet for 1 week significantly improved blood glucose control in mice (64, 65). Moreover, the glucose tolerance and pyruvate tolerance of mice fed a low-leucine diet were significantly increased, indicating that leucine restriction could enhance gluconeogenesis (41). Leucine can also regulate glucose uptake by L6 myotubes independent of the mTORc1 and AKT signaling pathways, and the concentration of leucine in the culture medium has a dose-dependent relationship with non-insulin-stimulated glucose uptake in cells (66). Nevertheless, low concentrations of leucine did not change insulin signaling and were not associated with insulin resistance but increased the lipid content in myotubes (67). A cohort study of women without prediabetes also showed that leucine supplementation had no effect on insulin sensitivity (68).

Recent studies have shown that valine supplementation alone has no effect on pAkt and Akt in myotubes and was not associated with insulin stimulation or different levels of insulin resistance (69). However, valine in combination with leucine, isoleucine or protein can affect insulin signaling and related metabolic pathology. Moreover, excessive intake of valine can independently induce insulin resistance. 3-Hydroxyisobutyric acid (3-HIB) is a catabolic intermediate of valine (70). In animals, 3-HIB is secreted by muscle, which activates the transport of endothelial fatty acids, stimulates the uptake of muscle fatty acids, and promotes muscle lipid accumulation and insulin resistance (8). The addition of 3-HIB to white and brown adipose cell cultures increased fatty acid uptake and regulated insulin-stimulated glucose uptake in a time-dependent manner (70). In humans, 3-HIB has been shown to be associated with insulin resistance in subjects with diabetes and with insulin resistance in overweight and obese individuals (71). It was shown that in a cohort of 4,942 men and women, cyclic 3-HIB increased with elevated levels of hyperglycemia and T2DM. In addition, after weight loss, cyclic 3-HIB concentrations increase briefly and then decrease significantly.

Studies of isoleucine have shown that it can significantly increase muscle and fat mass and cause insulin resistance. Furthermore, it can also upregulate the levels of key lipogenic proteins and myogenic proteins. More importantly, through mitochondrial function lesions, isoleucine can harm the gastrocnemius and tibialis anterior and lead to cavitation, swelling, cristae fractures, etc. In addition, isoleucine promotes myogenesis and increases lipid droplet accumulation in myotubes. In general, isoleucine can increase muscle mass and induce insulin resistance through myogenesis and intracellular lipid deposition (72).

Although most of the recent studies have confirmed that an appropriate supply of BCAAs to the normal body has no or a positive effect on insulin sensitivity, in the diabetic or obese body or in the case of an excessive supply of BCAAs to the normal body, BCAAs have negative effects on insulin sensitivity and sometimes can lead to insulin resistance. At present, there are several speculations about the mechanism of this phenomenon: one is the catabolite of BCAAs, that is, branched-chain ketone acids (BCKAs). The expression of BCAA catabolic enzymes in the hearts of fasting mice was decreased, and circulating BCKAs were increased, while BCAAs were not increased. Similar increases in circulating BCKAs were associated with changes in BCAA catabolic enzyme expression in diet-induced obesity (DIO) mice. Exposure of muscle cells to high levels of BCKAs inhibits insulin-induced AKT phosphorylation and reduces glucose uptake and mitochondrial oxygen consumption. Changes in the intracellular BCKA clearance rate by gene-regulated expression of BCKDK and BCKDHA have similar effects on AKT phosphorylation. Therefore, excessive amounts of BCKAs are the real cause of insulin resistance (73). Second, it has been hypothesized that mammalian target of rapamycin complex 1 (mTORC1) is overactivated in the presence of amino acid overload, leading to a reduction in insulin-stimulated glucose uptake, which is caused by insulin receptor substrate (IRS) degradation and reduced Akt-AS160 activity (56). However, this hypothesis can only explain the negative effects of excessive intake of BCAAs but cannot explain the regulatory mechanism of insulin sensitivity by the addition of BCAAs in disease models. Furthermore, the results still do not explain the findings on HMB (the metabolite of leucine – hydroxy-p-methylbutyric acid), which is thought to reduce insulin resistance (74). HMB may reduce insulin resistance and hepatic steatosis by inhibiting GLUT-2 in the liver of high-fat diet-fed rats (75). For humans, acute HMB treatment improves insulin resistance after glucose loading in young men but has no effect on insulin sensitivity in older men (76). The above phenomena are not consistent with the effect of leucine on insulin resistance, and there is no recent research on the mechanism of this contradiction, so the regulation of these phenomena requires further investigation.

## 4. Lifespan and insulin resistance

The improvement in insulin resistance is associated with longevity. The metabolic traits in centenarians are maintaining insulin sensitivity and a lower incidence of diabetes (77), which suggests that glucose homeostasis may play a crucial role in health and longevity. At present, much of the increase in longevity and health is achieved by improving insulin sensitivity. New insights into certain features of vegetarianism suggest that vegetarianism can improve insulin resistance and dyslipidemia and related abnormalities by limiting proteins or certain amino acids (leucine or methionine) and has the potential to extend life (78). Aging PASK-deletion mice exhibited overexpression of the longevity gene FoxO3a and a normal HOMA-IR index, which simultaneously confirmed that mice lacking PASK had better insulin sensitivity and glucose tolerance (79). *Drosophila melanogaster* fed a high-sugar diet showed signs of insulin resistance and a reduced lifespan (80). Moreover, in mice and humans, an increase in circulating BCAAs is associated with a high risk of insulin resistance and diabetes, as well as an increased mortality in mice (31). Studies of calorie restriction (CR) have shown that eating high-calorie foods can impair metabolism and accelerate aging; in contrast, CR can prevent age-related metabolic diseases and extend life (81), and the main mechanism by which calorie restriction prolongs life is to improve aging by enhancing insulin sensitivity (82, 83), which also suggests a positive correlation between increased longevity and insulin sensitivity. In a study of mice fed a medium-fat or high-fat diet, it was observed that low insulin levels significantly increased lifespan, high insulin levels contributed to age-dependent insulin resistance, and decreased basal insulin levels could extend lifespan (84). These results suggest that increased insulin levels and insulin sensitivity have a positive effect on longevity.

Although several studies have shown that improved insulin sensitivity promotes health and longevity, there is also clear evidence that insulin sensitivity is not necessarily related to healthy aging and may even be counterproductive. In some insulin-related gene knockout mouse models, insulin resistance is induced in one or more tissues, and a significant increase in the lifespan of mice can be observed. The average lifespan of mammalian insulin-like growth factor type 1 receptor (IGF-1R) knockout mice was 26% longer than that of wild-type mice, and the knockout mice showed stronger antioxidant stress resistance (85). Fat-specific insulin receptor knockout (FIRKO) in both male and female mice increased the average lifespan by approximately 134 days (18%) (14). Female insulin receptor substrate (IRS) 1 knockout mice also showed signs of longevity (15). Furthermore, rapamycin extended the lifespan of mice by inhibiting mTORC1 and impaired glucose homeostasis to induce insulin resistance by interfering with mTORC2 signaling (86). S6K1 knockout female mice showed an extended lifespan and induced loss of insulin sensitivity (33).

**Figure 3:**
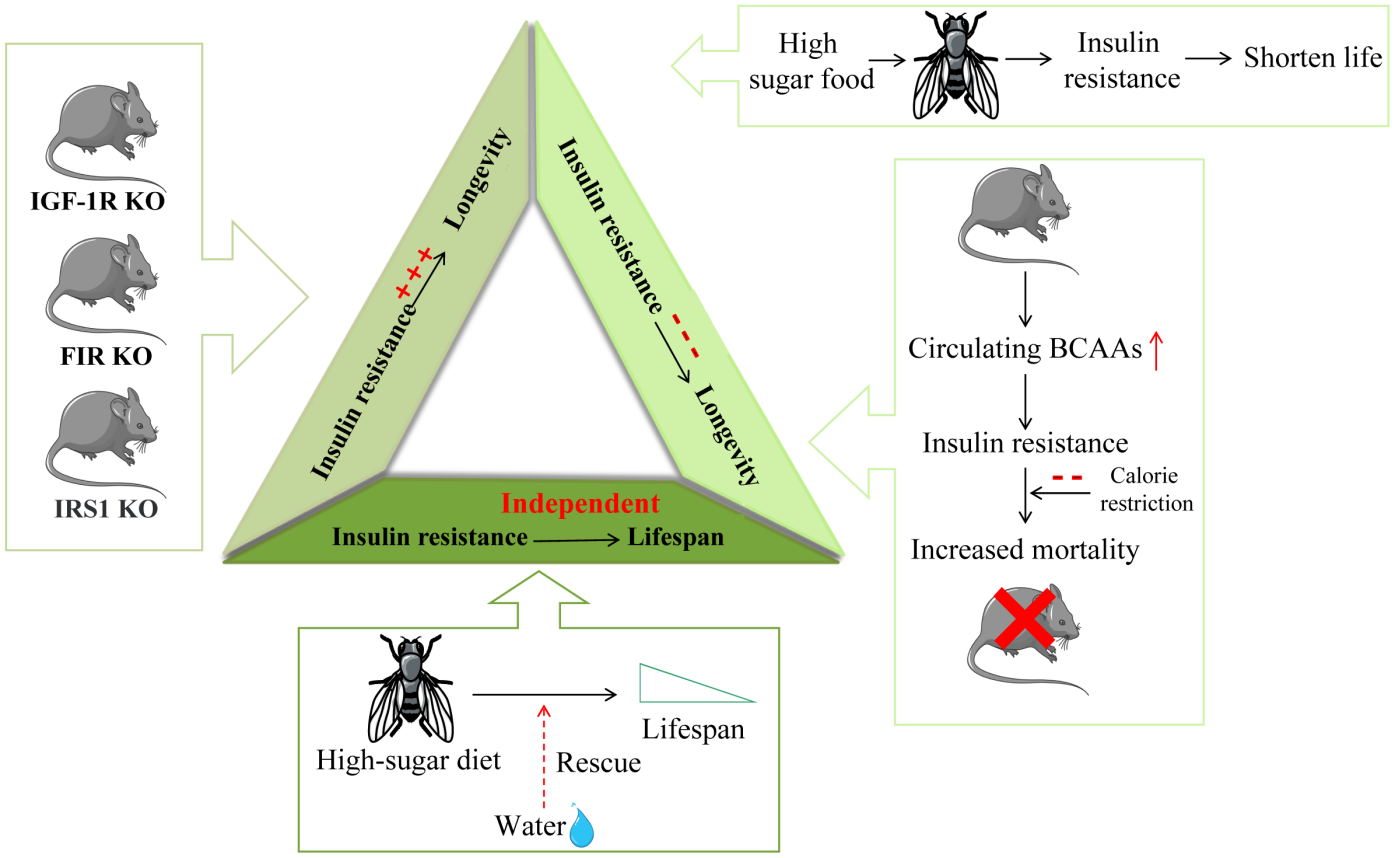
The different relationship between insulin resistance and longevity. Insulin resistance in insulin-related gene IGF-1R, FIR, and IRS1 knockout mice is induced in one or more tissues and is accompanied by a significant extension of mouse lifespan. On the other hand, Drosophila fed a high-sugar diet are characterized by insulin resistance and a shortened lifespan. Increased circulating BCAAs in mice lead to insulin resistance development and increased mortality in mice. Furthermore, calorie restriction (CR) can extend life by improving insulin sensitivity. Moreover, the shortened lifespan caused by high sugar feeding in Drosophila can be saved by water, suggesting that insulin resistance may be independent of lifespan regulation.

Previous studies have focused on the positive and negative correlations between insulin resistance and lifespan, but recent findings suggest the more interesting possibility that insulin resistance or metabolic defects may be independent of longevity, which also explains the above two opposite results. As insulin resistance is independent of lifespan, the opposite results of lifespan may be led by other different factors, and insulin resistance is not the main reason for lifespan-changing. From this study we could see that a high-sugar diet can shorten the lifespan of Drosophila, which could be saved by water supplementation. In contrast, metabolic defects, which are widely thought to lead to reduced survival, have been shown to be unrelated to water. Drosophila that had been watered on a high-sugar diet still showed all the metabolic defects similar to diabetes and had the same survival rates as healthy controls, suggesting that obesity and insulin resistance by themselves did not shorten fly life. The mechanism by which a high-sugar diet regulates lifespan in Drosophila is thought to be a water-dependent way of regulating uric acid production: the high-sugar diet promotes the accumulation of uric acid (a final product of purine catabolism) and enhances this process. It is obviously shown that renal calculi occur on a 20% sucrose diet, with impaired renal tubule secretion and impaired purine metabolism leading to uric acid accumulation in the urinary cavity. Furthermore, this phenomenon is completely restored by water or allopurinol treatment (87). Mice deficient in fatty acid binding protein (FABP) showed extended metabolic health cycles, which were protective against insulin resistance, glucose intolerance, inflammation, deterioration of adipose tissue integrity, and fatty liver disease. However, the mice lacking FABP showed no signs of longevity. These data suggest that metabolic health in mice can be detached from longevity in the absence of caloric restriction, suggesting that these pathways may act independently (88) (Figure 3).

## 5. Conclusion

This review summarizes the contradictory role of branched-chain amino acids in lifespan and insulin resistance. In this review, we attempt to explain these conflicting findings from a physiological and pathological perspective and to draw conclusions about the possible regulatory mechanisms between BCAAs and aging, BCAAs and insulin resistance, and aging and insulin resistance. When explaining the above arguments, it should be noted that (1) metabolic regulation of the body is very complex, and the existence of aging and insulin resistance may also have an impact on metabolism, including some compensatory effects; (2) the effects of BCAA supplementation on aging and insulin resistance should be comprehensively analyzed in combination with sex, age, dietary conditions and basic diseases; (3) at present, exogenous BCAAs are not well correlated with endogenous BCAA levels in plasma and serum; and (4) there are some great differences in diseases, aging, food intake and water intake, especially in animal models. Therefore, BCAA-based research must refer to the basic survival data of the research subject for comprehensive analysis. Due to the complexity of metabolism and body responses, it is difficult to draw convincing conclusions about the effects of BCAAs on aging and insulin resistance.

From the screening of the literature, the following possible regulatory mechanisms of BCAAs, aging and insulin resistance can be concluded: (1) Autophagy can significantly extend the lifespan of yeast, *C. elegans* and mice. However, autophagy alone is neither sufficient to extend the lifespan nor necessary to extend the lifespan. In the absence of autophagy, the inhibition of protein synthesis in animals that were fed adequate food could extend life. In summary, autophagy may have a specific regulatory mechanism to prolong life, and its specificity may be related to the environment of the food supply and the regulation and triggering of longevity pathways. To some extent, this also explains the mechanism by which BCAAs and insulin resistance influence lifespan. However, there is currently no reliable study on the triggering mechanism by which insulin resistance affects autophagy, and the study examining the trigger of autophagy by BCAAs also shows contradictory results. This suggests that a certain limit of autophagy may have a positive effect on the regulation of lifespan, while excessive autophagy may have a negative effect, but there is no clear conclusion as to what the limit is. (2) Endogenous BCAAs have a significant influence on lifespan. Although there is no exact correlation between endogenous and exogenous BCAA levels at present, the conclusion that endogenous BCAA accumulation caused by diseases such as obesity and T2DM leads to aging and damage to the body has been very clear. Therefore, the negative effects of BCAAs on the body based on insulin resistance and various diseases can be explained. However, elderly individuals have low concentrations of BCAAs in the plasma, but this was not the case in young individuals and children (89, 90). Therefore, the positive effects of BCAA supplementation on aging can also be explained. However, theories based on endogenous BCAAs cannot explain the positive effects of insulin resistance on longevity. To date, many hypotheses have been proposed based on these contradictions. However, there are various problems, such as the lack of credible evidence and the inability to explain some of the results. Therefore, there are no credible theories about the regulatory mechanisms of BCAAs, aging and insulin. However, two conclusions are convincing: one is that BCAAs remain an effective supplement for aging and related metabolic changes, and the other is that maintaining endogenous BCAAs within a reasonable range is indeed important for health. Therefore, there is still a long way to go to further explore the relationship between BCAAs, longevity and insulin resistance based on existing research.

Besides, the metabolites of BCAAs have been the focus of attention in recent years. Studies were no longer limited to BCAAs study on lifespan and insulin resistance. Studying the separate amino acids of BCAAs and the metabolites of BCAAs is on the rise. This can further research the mechanism of BCAA-regulated metabolic disorders in the body. In this review, we mainly talked about the metabolite of valine – 3-HIB. This metabolite could lead to the activation of fatty acid transportation in some organs, which is related to insulin resistance. After that, the insulin resistance was induced in the body. In summary, the 3-HIB is a promising marker for detecting insulin resistance. However, as the essential factor of BCAAs, leucine has fewer reports about its metabolites that have an effect on insulin resistance. So we have reason to believe that with the discovery of more metabolites of BCAAs that are in association with insulin resistance, the mechanism of BCAAs and insulin resistance will be more promising. And these metabolites will be the potential targets to treat insulin resistance in T2DM.

In conclusion, recent studies suggest that endogenous BCAAs, BCAA metabolism and mTOR-related autophagy play important roles in the relationships among BCAAs, longevity, and insulin resistance. The recent discovery that insulin resistance may be independent of longevity has expanded our understanding of the regulatory mechanisms among the three. However, there is still no definite conclusion on the specific conditions under which BCAAs and insulin resistance extend life, shorten life, or do not change lifespan, and there is still no credible and comprehensive explanation for the different effects of BCAAs and insulin resistance on lifespan. In addition, similar confusion occurs between BCAAs and insulin resistance. These problems are attracting increasing research interest, and the study of these problems is conducive to the elucidating the rational use of BCAAs, identifying a treatment for T2DM and the study of longevity.

## Author contributions

YaL and YiL supervised the entire project. HY drafted the manuscript and prepared the figures. KL and JW revised the manuscript. All authors have read and agreed to the published version of the manuscript.

## Funding

This work was supported by grants from the CAMS Innovation Fund for Medical Sciences (No. 2018-I2M-1-002) and the National Natural Science Foundation of China (No. 81671391).

## Conflict of interest

The authors declare that the research was conducted in the absence of any commercial or financial relationships that could be construed as a potential conflict of interest.

## Publisher’s note

All claims expressed in this article are solely those of the authors and do not necessarily represent those of their affiliated organizations, or those of the publisher, the editors and the reviewers. Any product that may be evaluated in this article, or claim that may be made by its manufacturer, is not guaranteed or endorsed by the publisher.
